# Decreased levels of cathepsin Z mRNA expressed by immune blood cells: diagnostic and prognostic implications in prostate cancer

**DOI:** 10.1590/1414-431X2021e11439

**Published:** 2021-08-06

**Authors:** A.A.S. Batista, B.M. Franco, M.M. Perez, E.G. Pereira, T. Rodrigues, M.L. Wroclawski, F.L.A. Fonseca, E.R. Suarez

**Affiliations:** 1 Centro de Ciências Naturais e Humanas, Universidade Federal do ABC, Santo André, SP, Brasil; 2 Laboratório de Análises Clínicas, Centro Universitário Faculdade de Medicina do ABC, Santo André, SP, Brasil; 3 Hospital Israelita Albert Einstein, Santo André, SP, Brasil; 4 Departamento de Ciências Farmacêuticas, Universidade Federal de São Paulo, Diadema, SP, Brasil

**Keywords:** Cathepsin X, Cathepsin K, Prostate-specific antigen, Biopsy, Biomarker, Prostate cancer

## Abstract

Cathepsin Z (CTSZ) is a cysteine protease responsible for the adhesion and migration of both immune and tumor cells. Due to its dual role, we hypothesized that the site of CTSZ expression could be determinant of the pro- or anti-tumorigenic effects of this enzyme. To test this hypothesis, we analyzed CTSZ expression data in healthy and tumor tissues by bioinformatics and evaluated the expression levels of CTSZ mRNA in the blood cells of prostate cancer (PCa) patients by qRT-PCR compared with healthy subjects, evaluating its diagnostic and prognostic implications for this type of cancer. Immune cells present in the blood of healthy patients overexpress CTSZ. In PCa, we found decreased CTSZ mRNA levels in blood cells, 75% lower than in healthy subjects, that diminished even more during biochemical relapse. CTSZ mRNA in the blood cells had an area under the curve for PCa diagnosis of 0.832, with a 93.3% specificity, and a positive likelihood ratio of 9.4. The site of CTSZ mRNA expression is fundamental to determine its final role as a protective determinant in PCa, such as CTSZ mRNA in the blood cells, or a malignant determinant, such as found for CTSZ expressed in high levels by different types of primary and metastatic tumors. Low CTSZ mRNA expression in the total blood is a possible PCa marker complementary to prostate-specific antigen (PSA) for biopsy decisions, with the potential to eliminate unnecessary biopsies.

## Introduction

Prostate cancer (PCa) is the second most incident type of cancer worldwide and the first in the male population, representing the second leading cause of male cancer death in the United States (1). Prostate-specific antigen (PSA) is a glycoprotein widely used as a molecular marker for PCa screening due to its high sensitivity (2,3). PSA testing improved PCa screening; however, the specificity, positive likelihood ratio (+LR), and positive predictive value are low (2,3), and approximately one-third of the men with an elevated PSA have PCa detected on biopsy. These data raise concerns since numerous patients are submitted unnecessarily to the risks and discomforts of a prostate biopsy, such as bleeding, infection, and pain.

Cathepsin Z (CTSZ), also known as X or P, is a differentiated member of the cathepsins that has only mono- and dipeptidyl carboxypeptidase activity, being responsible for cleavage and activation of some molecules, such as the integrin called lymphocyte function-associated antigen (LFA-1), the focal adhesion kinase (FAK), the SRC kinase, and others. The cleavage of LFA-1 changes cell morphology, promoting cell migration (4). Still, the RNAi silencing of CTSZ in T cells remarkably decreases its spreading, demonstrating the importance of this enzyme for the function of this cell type (5). The interaction between T cells and endothelial cells of blood vessels can coordinate T cells' efficient circulation in the tumor microenvironment. CTSZ can improve adhesion and the consequent migration of T cells through the endothelia, increasing the levels of tumor-infiltrated CD8 T cells, a well-known marker of good prognosis and survival in patients with several types of cancer (6). Immune cells, such as monocytes, macrophages, and dendritic cells, regularly express CTSZ (7). CTSZ is overexpressed in several primary tumor types, such as PCa, colorectal, gastric, liver, melanoma, and pancreatic neuroendocrine tumors (4). In some of these studies, CTSZ was found in tumor-associated macrophages, indicating that this protease plays a role in the antitumor immune response. In all these cases, the CTSZ relationship with integrin receptors is fundamental to allow cell dissemination through the extracellular matrix (7).

Here, we analyzed CTSZ expression in healthy and tumor tissues and evaluated the diagnostic and prognostic implications of CTSZ mRNA expressed by blood cells in prostate cancer.

## Material and Methods

### Bioinformatics data

We selected the RNA-seq data of human CTSZ mRNA expression from the Human Protein Atlas (HPA) RNA-seq normal tissues project (https://www.proteinatlas.org/), the Genotype-Tissue Expression (GTEx) Project (8), and from the Pan-Cancer Analysis of Whole Genomes (https://dcc.icgc.org/pcawg). The bioinformatics data presented herein are available at the Expression Atlas website (9). For the HPA data, the RNA-seq was performed on tissue samples from 95 people representing 27 different tissues to determine tissue-specificity of all protein-coding genes (BioProject accession number PRJEB4337 in the DDBJ BioProject database (10). The GTEx Project has the RNA-seq from 53 human tissue samples (8), and the Pan-Cancer Analysis of Whole Genomes has RNA-seq data of more than 2800 cancer patients, including 20 different primary sites. We built a heat map and a graph of CTSZ mRNA expression from healthy and cancer tissues to compare their mRNA expression in TPM (transcripts per kilobase million). The CTSZ mRNA expression in different immune cells from the human blood was obtained from the Human Protein Atlas Website (https://www.proteinatlas.org) (11).

### Patients and study design

This cross-sectional retrospective pilot study included thirty patients with PCa and twenty healthy male subjects from the Urologic Oncology Clinic at the Faculdade de Medicina do ABC, Brazil. As inclusion criteria for the case group, we selected patients with PCa diagnosis, without previous treatment, and over 18 years of age. All subjects had undergone 12-core transrectal ultrasound-guided prostate biopsy due to elevated PSA levels or abnormal digital rectal exam. The exclusion criteria were other associated comorbidities that could influence the inflammatory status, such as autoimmune diseases, allergies, and infections. The control group inclusion criteria were a negative personal, first, or second-degree family history of cancer, negative PSA (≤2.5 ng/mL, in accordance with the American Cancer Society Recommendation for Prostate Cancer Early Detection), and carcinoembryonic antigen (CEA). The PSA distribution in the control and case group is presented in Supplementary Figure S1. The FMABC Institutional Review Board approved this research (CAAE: 57559516.3.0000.0082), and all participants included in this study signed informed consent. Blood samples were collected with EDTA as an anticoagulant, before and six months after the different treatments, according to disease stage: radical prostatectomy or radiotherapy plus androgen deprivation therapy (ADT) (for localized PCa) and ADT (for advanced PCa). Treatment was chosen according to clinical criteria. A more detailed schematic representation of the study design is presented in Supplementary Figure S2.

The CTSZ expression was analyzed and compared to the following clinical-pathological characteristics: ISUP (International Society of Urological Pathology) grade group on biopsy, ISUP grade group on the tumor, clinical stage, type of treatment, pathologic stage, tumor margin positives, biochemical relapse, and death by cancer. For the analysis of the ISUP grade group for biopsy or tumor, we divided the samples into three groups: a low-risk group, constituted by patients with ISUP score of 1, an intermediate-risk group for patients with ISUP score of 2 or 3, and a high-risk group for ISUP score of 4 or 5. For the clinical-stage, we subdivided the patients into three groups: localized disease (T1N0M0 or T2N0M0), locally advanced (T3N0M0), or metastatic disease (TXNXM1). We grouped the patients according to the types of treatment performed as radical prostatectomy (P), hormone therapy (H), and radiotherapy plus hormone therapy (R+H). For the pathological stage, T2N0orX had the tumor confined inside the prostate, T3aN0orX had extra-capsular involvement of the tumor, and T3bN0orX were tumors presenting invasion of the seminal vesicle. The tumor margin was considered negative (free) or positive (committed), and the occurrence of biochemical relapse or death by cancer was designated as yes or no. We defined biochemical recurrence whenever the PSA level surpassed 0.2 ng/mL after prostatectomy or a rise of 2 ng/mL or more above the lowest PSA levels after radio-hormonal therapies.

### RNA extraction, RT-PCR, and real-time PCR (qPCR)

RNA from total blood was extracted using Trizol LS (GE Healthcare, USA) following the manufacturer's instructions. The RNA was submitted to reverse transcription using the ImProm-II (TM) Reverse Transcription System kit (Promega, USA). *CTSZ* and housekeeping genes were amplified using 2 µL of cDNA of each sample previously diluted 1:15, 5 µL of Power SYBR Green Master Mix (2X) (Thermo Scientific, USA), and 1.5 µL of primers sense and antisense (6 µM). The samples were amplified in the Applied Biosystems^®^ 7500 Real-Time PCR System (Life Technologies, USA) for 40 cycles of 15 s at 95°C, followed by 60 s at 60°C. The following primer sequences were used: *CTSZ* sense: 5′ CATCCCTGACGAGACCTG 3′, *CTSZ* antisense: 5′ GCATGTCCCACATTGGTTAAA 3′; ribosomal protein 13a (*RPL13a*) sense: 5′ CCACCCTGGAGGAGA 3′ and *RPL13A* antisense: 5′ CCTGTTTCCGTAGCCTCAT 3′. All experiments were performed in triplicate for each sample. CTSZ mRNA expression was firstly normalized by the housekeeping gene RPL13a (2^-ΔCt^) and then corrected by the CTSZ mRNA expression from the healthy control group (2^-ΔCt^). RPL13a was previously used to correct CTSZ expression from the control group.

### Statistical analysis

The statistical significance of the data was analyzed using the IBM SPSS Statistics 20 software (IBM^®^ Inc; USA). The variables were analyzed for normality by the Kolmogorov-Smirnov test. Mann-Whitney or Kruskal-Wallis tests were used to compare non-parametric variables of two or more than two groups, respectively. A *t-*test or ANOVA followed by a Bonferroni post-test was applied to compare parametric variables of two or more than two groups, respectively. We considered a two-tailed analysis and a significance level of 0.05. The ROC curve was calculated for CTSZ mRNA in the blood as a molecular marker for PCa diagnoses or biochemical recurrence.

## Results

### Comparative analysis of CTSZ mRNA levels in healthy and primary cancer tissues

**Figure 1:**
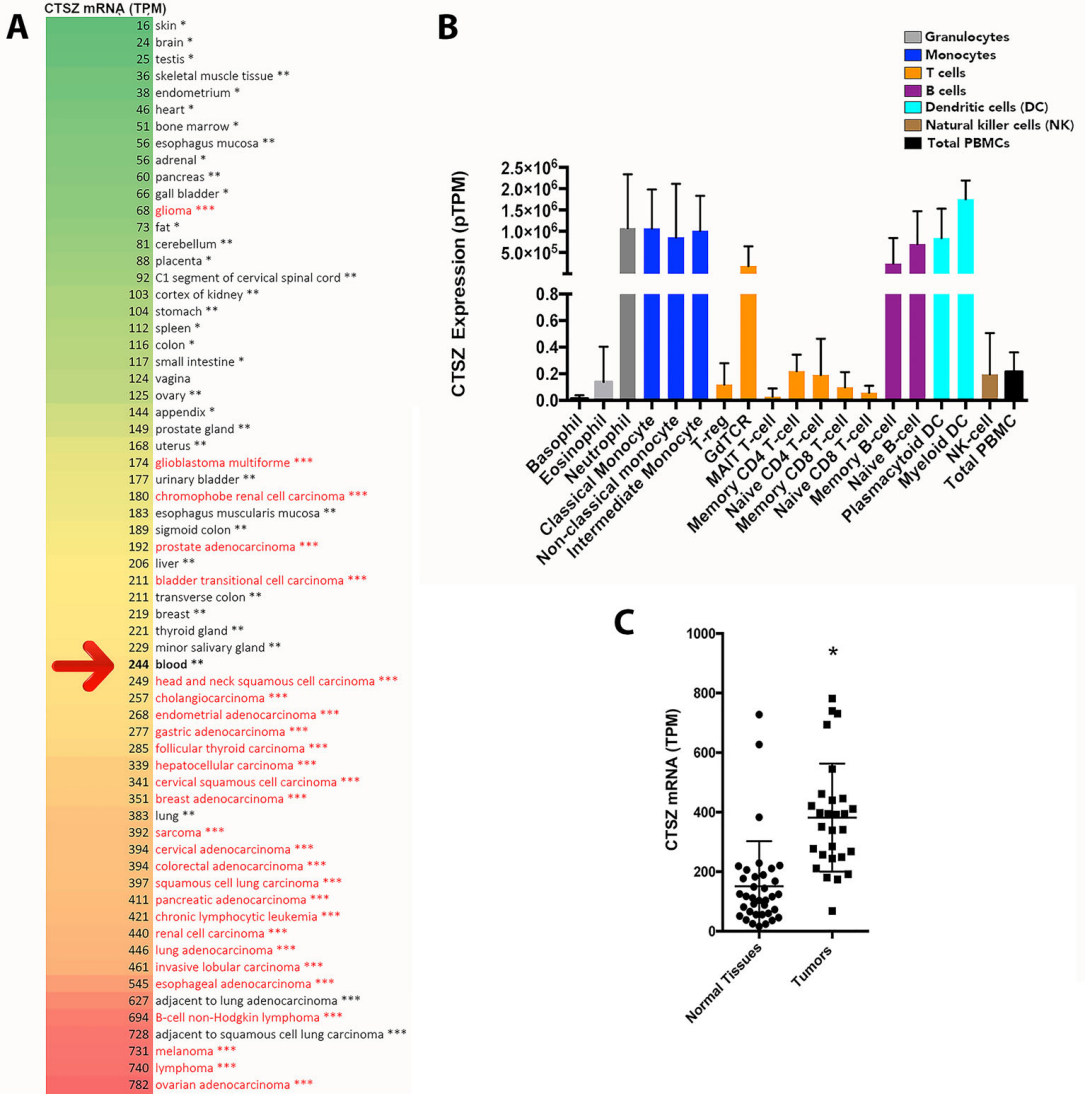
Bioinformatics data of cathepsin Z (CTSZ) expression. **A**, Heat map of CTSZ mRNA expression from several normal and cancer tissues. The red arrow indicates the expression of CTSZ mRNA in the blood of healthy subjects. Black font: healthy tissues; Red font: tumors. *RNA-seq mRNA expression from the HPA RNA-seq normal tissues project (https://www.proteinatlas.org), **RNA-seq data from the Genotype-Tissue Expression (GTEx) Project, and ***RNA-seq m from the Pan-Cancer Analysis of Whole Genomes (https://dcc.icgc.org/pcawg, data available in the Expression Atlas website). TPM: transcripts per kilobase million. **B**, CTSZ mRNA expression in different human immune cells from healthy subjects' blood. **C**, CTSZ mRNA expression in normal tissues versus tumors. *P<0.05 (*t*-test). We adapted the data presented in panel B from the Human Protein Atlas website (https://www.proteinatlas.org).

We first provided a comparative overview of the CTSZ mRNA expression in different tumor and non-tumor sites in the body, including the whole blood of healthy patients, analyzing RNA-seq data of CTSZ mRNA expression in large cohorts of subjects (Figure 1). Interestingly, we found that the blood of healthy subjects presented high CTSZ mRNA levels (Figure 1A). We can note in Figure 1B that the expression of CTSZ mRNA was very high in the immune blood cells of healthy patients. These data associated with the CTSZ known function of promoting immune cell adhesion, with consequent migration through the endothelia to solid tissues, suggest a possible protective potential against cancer development. In solid tissue sites, all healthy tissues expressed less CTSZ than malignant tumors, except low-grade gliomas (Figure 1A and B, P=0.010). The intense expression of CTSZ mRNA in malignant tumor sites can be explained, at least in part, by the immune infiltration that often occurs in cancer tissues. This can be explained by the fact that regular sites adjacent to tumors and a few specific sites that have, in common, high levels of mucosa-associated lymphoid tissue/immune system cells (e.g., lung, blood, salivary glands, thyroid, breast, transverse/sigmoid colon, and esophagus) also have high levels of CTSZ (Figure 1A).

Since no data about the concentration of CTSZ mRNA in the total blood of cancer patients were available in the databases evaluated, we decided to explore this in a pilot clinical study based on blood samples of PCa patients.

### CTSZ mRNA expression in the blood as a predictive factor for PCa diagnosis

**Figure 2:**
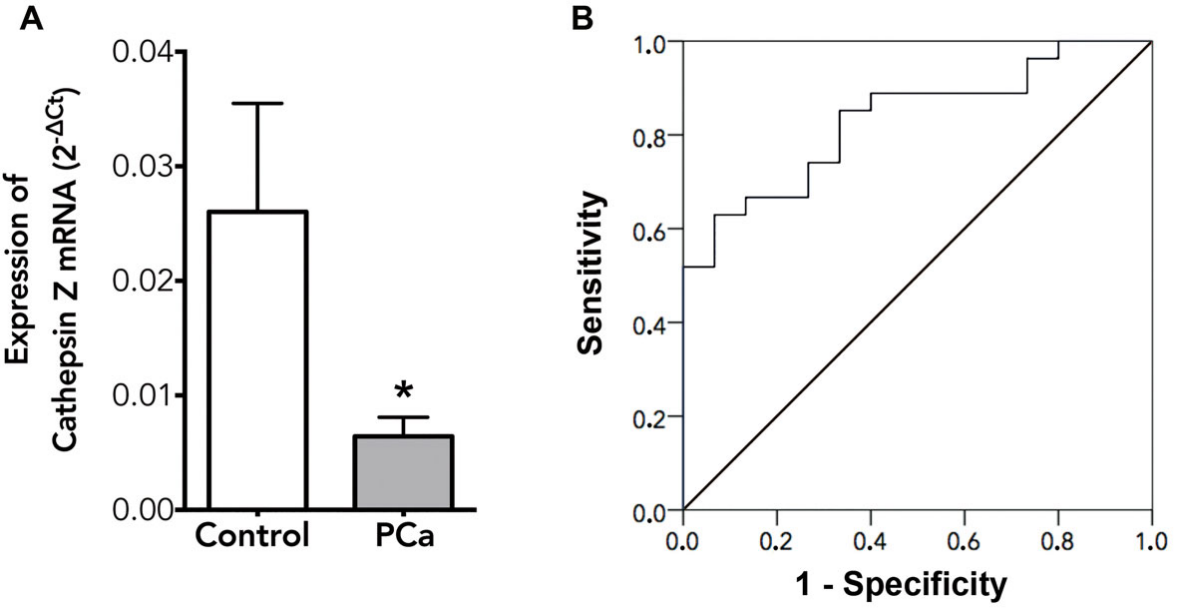
**A**, Relative expression of cathepsin Z (CTSZ) mRNA in the blood of patients with prostate cancer (PCa) before treatment compared with healthy subjects (control) determined by real-time polymerase chain reaction (qPCR). Data are reported as means±SD. *P=0.002 (*t*-test). **B**, Receiver operating characteristic (ROC) curve of CTSZ mRNA as a molecular marker of PCa, with an area under the curve (AUC) of 0.832±0.062, P<0.001.

Table 1 shows the relative and the absolute number of cases by each clinical-pathological feature analyzed. The relative expression of CTSZ mRNA in the total blood of patients with PCa before treatment was 75% lower than the CTSZ mRNA expression observed in the control group (Figure 2A; P=0.002). This variable was considered as a good predictive factor for the diagnosis of PCa, as determined by the ROC curve analysis (Figure 2B), which presented an area under the curve (AUC) of 0.832±0.062 (95%CI: 0.711-0.953), P<0.001. This wide confidence interval indicates an interference of the sample size of this pilot study, however even with this limitation, we can note that the lowest value of the CI is higher than 0.7, reinforcing CTSZ mRNA as a good marker for PCa diagnosis. The estimated sensitivity of a CTSZ mRNA is 63% for PCa detection, but this test's specificity is high, achieving 93.3% considering a cut-off <0.0049. Furthermore, the CTSZ mRNA positive likelihood ratio (+LR) is 9.4, while the negative likelihood ratio (-LR) is close to 0.4.

**Table 1:** The relative and the absolute number of patients by clinical-pathological features.

	Sample size (N)	Percentage (%)
Control group	20	40
Prostate cancer patients	30	60
All cases	50	100
Age^*^ (years)		
Control group	69±7	N/A
Prostate cancer group	66±8	N/A
Race** (Control group/ Prostate cancer group)		
White	9/12	45/40
Pardo^†^	8/10	40/34
Afro-American	3/7	15/23
Asian	0/1	0/3
ISUP grade biopsy - Risk		
Low (=1)	11	36.7
Intermediate (=2 and 3)	13	43.3
High (≥4)	6	20.0
Clinical Stage		
Localized	26	86.7
Locally advanced	2	6.7
Metastatic	2	6.7
Treatment		
Prostatectomy	20	66.7
Hormonal therapy	6	20.0
Radio + hormonal therapy	4	13.3
Pathologic Stage		
T2N0orX	13	43.3
T3aN0orX	4	13.3
T3bN0orX	3	10.0
N/A	10	33.3
ISUP grade tumor - risk		
Low (=1)	7	23.3
Intermediate (=2 and 3)	11	36.7
High (≥4)	2	6.7
N/A	10	33.3
Tumor margin		
Negative	14	46.7
Positive	6	20.0
N/A	10	33.3
Biochemical relapse		
No	20	83.3
Yes	4	16.7
Death by cancer		
No	18	60.0
Yes	3	10.0
N/A	9	30.0

P=0.215 for age; N/A: Not applicable; **P=0.728 for race; ^†^mixed Afro-American and White race. ISUP: International Society of Urological Pathology.

### Clinical pathological features and CTSZ mRNA levels in the blood

We have also analyzed CTSZ mRNA expression in terms of different clinical parameters related to the malignancy status, and we found that the group with intermediate/high risk according to the ISUP grade group for the resected tumor (Supplementary Figure S3A) presented a 71% decrease compared to the low-risk group (p=0.050). Furthermore, patients with biochemical relapse had a 54% decrease in the CTSZ mRNA expression (P=0.024) compared with PCa patients with no relapse (Supplementary Figure S3B). For this relationship, we calculated a ROC curve, which presented an AUC of 0.763 (95%CI: 0.546-0.979), but it was not statistically significant (P=0.104). For the other parameters analyzed (Supplementary Figure S3C-H) and for the patients submitted to prostatectomy, where we evaluated the expression of CTSZ mRNA in the blood before and six months after the surgical excision of the tumor, no significant difference was found in CTSZ mRNA expression among the groups (Supplementary Figure S4). It is important to point out that both control and case groups were statistically homogeneous in terms of age (P=0.215) and race (P=0.718).

## Discussion

Considering the current markers used for PCa diagnosis, PSA is still the gold standard for PCa screening in men. PSA is regarded as a high sensitivity test for PCa diagnosis in most studies, varying from 67 to 90%; however, this test's specificity is low, ranging from 28 to 59% (2,3,12). CTSZ mRNA can be considered as a molecular marker with similar sensitivity to PSA, but superior specificity for PCa diagnosis, at least in the population analyzed in this study. The +LR for CTSZ mRNA levels <0.0049 was 9.4, which means that it is 9.4 times more common in men with PCa than in those without the disease, increasing the probability of PCa by approximately 45%. For comparison purposes, +LR for PSA is only 1.28 (2), which means that a positive test increases the disease's probability by less than 15%. The -LR for CTSX mRNA is very similar to PSA, being close to 0.4 (2), which means that a negative result (CTSZ mRNA >0.0049) decreases the PCa probability by almost 20%. Since clinical decisions are guided by estimations of the likelihood of disease, decisions derived from interval LR use may be more informative than sensitivity and specificity. The comparison between CTSZ mRNA and PSA performance as molecular markers for PCa detection must be evaluated with caution since this pilot study had a limited number of patients, while PSA data used for comparison were collected from several large clinical trials. It is interesting to note that the AUC for CTSZ mRNA is higher than most published AUC for markers in PCa diagnosis, including PSA, the 4-kallikrein panel, Prostate Health Index (PHI), multiparametric magnetic resonance imaging (mpMRI), and the association of PHI and mpMRI. However, the superior performance of CTSZ mRNA in PCa diagnosis observed in this study may have been influenced by the reduced sample size of this pilot study, associated with its retrospective nature, with the absence of a biopsy performance in the control group and the use of PSA as the leading guide for the pre-diagnostic selection of this group. Therefore, future large prospective cohort trials must be designed to evaluate CTSZ mRNA as well as CTSZ protein expression as potential diagnostic biomarkers for PCa. In these studies, a vital inclusion criterion would be a negative biopsy result for the control group members. With this in mind, we can summarize that CTSZ mRNA expression (2^-ΔCt^) is a molecular marker of high specificity and +LR when compared with PSA. However, CTSZ mRNA has a similar sensitivity and -LR compared to PSA. Thus, CTSZ mRNA would be helpful as a complement of PSA to confirm the positive diagnosis of PCa in patients with a slightly high PSA, or cases of a suspicious digital rectal exam with normal PSA, with the potential to eliminate the necessity of biopsy in many instances, avoiding its associated risks, such as bleeding, infection, discomfort, and pain.

Low levels of CTSZ mRNA were found in PCa patients' blood in general and even lower levels were found in patients who had a biochemical relapse. In healthy subjects, this enzyme is expressed in high levels by specific immune cells, such as B cells, monocytes, neutrophils, and dendritic cells, highlighting the functional importance for T cell migration (5). T cells expressing CTSZ can infiltrate better into the tumor microenvironment through LFA-1 modulation, increasing the number of functional tumor-infiltrating lymphocytes (TILs) that could improve cytotoxic activity, contributing to avoid biochemical relapse and consequent progression of prostate tumors. If CTSZ is not expressed in high levels by immune cells from the blood, they do not achieve tumor sites efficiently, and the response against cancer cells can be impaired. The favorable prognostic value of cytotoxic (CD8) TILs has been demonstrated in multiple solid tumor types (13), including PCa (14,15).

Despite the widely available data regarding CTSZ mRNA levels in different types of primary malignant tumors, the information about CTSZ mRNA expression in the whole blood of cancer patients is limited. It is known that CTSZ protein levels in the serum of patients with inflammatory breast cancer are lower than in healthy subjects (16). However, for colorectal cancer patients, the serum levels did not differ from the control group (17). CTSZ protein levels in serum were significantly higher for lung cancer than in the healthy control subjects and presented short overall survival rates (18). None of these studies have used total blood as a sample, and none of them analyzed CTSZ mRNA, which hinders a proper comparison of the data. It must be determined if the CTSZ mRNA downregulation in the blood is a feature explicitly observed for PCa patients or if it can also occur in the total blood of patients with different tumor types.

PCa presents itself with both aggressive and indolent forms. Despite the controversy surrounding its use, PSA screening ultimately leads to a more significant number of diagnosed patients. One of the biggest challenges in clinical practice is selecting suitable patients for biopsy and, among diagnosed patients, differentiating tumors with an indolent course from those with an unfavorable prognosis to determine the best therapeutic decision for each case, avoiding unnecessary interventions. Currently, several types of biomarkers are available for clinical use in patients with PCa, which include blood-based tests (prostate-specific antigen, Prostate Health Index^®^, 4K score^®^), urine sample-based tests (PCA3, SelectMDx^®^, ExoDx Prostate IntelliScore^®^), and tissue-based tests (ConfirmMDx^®^, Oncotype^®^, Prolaris^®^, Decipher^®^) (19). However, although promising, the routine clinical use of PCa biomarkers still lacks strong evidence to be incorporated in the daily practice of the majority of urologists and oncologists. CSTZ may add necessary information to this scenario, and it has the potential to be included in biomarker tests developed in the future.

Our findings suggested that the body site of CTSZ mRNA expression was fundamental to determine its final role as a protective factor against cancer, such as CTSZ mRNA in blood cells, or a malignant determinant, such as found for CTSZ expressed in high levels by different types of primary and metastatic tumors. CTSZ mRNA expression in the blood was a molecular marker of high specificity and +LR for PCa in our cohort, with the potential to be helpful as a complementary biomarker to PSA to improve biopsy decision accuracy. The mechanisms regulating the expression of CTSZ mRNA in the blood of PCa patients still must be elucidated.

## Supplementary Material

Click here to view [pdf].
